# Mutations in the LKB1 tumour suppressor are frequently detected in tumours from Caucasian but not Asian lung cancer patients

**DOI:** 10.1038/sj.bjc.6604469

**Published:** 2008-07-01

**Authors:** J P Koivunen, J Kim, J Lee, A M Rogers, J O Park, X Zhao, K Naoki, I Okamoto, K Nakagawa, B Y Yeap, M Meyerson, K-K Wong, W G Richards, D J Sugarbaker, B E Johnson, P A Jänne

**Affiliations:** 1 Lowe Center for Thoracic Oncology, Dana-Farber Cancer Institute, Boston, MA, USA; 2 Department of Medical Oncology, Dana-Farber Cancer Institute, Harvard Medical School, Boston, MA, USA; 3 Department of Thoracic Surgery, Samsung Medical Center, Seoul, Korea; 4 School of Medicine, Sungkyunkwan University, Seoul, Korea; 5 Department of Medical Oncology, School of Medicine, Kinki University, Osaka, Japan; 6 Department of Medicine, Massachusetts General Hospital, Boston, MA, USA; 7 Department of Pathology, Harvard Medical School, Boston, MA, USA; 8 The Broad Institute of MIT and Harvard Universities, Cambridge, MA, USA; 9 Department of Medicine, Brigham and Women’s Hospital and Harvard Medical School, Boston, MA, USA; 10 Department of Surgery, Brigham and Women’s Hospital, Boston, MA, USA

**Keywords:** carcinoma, non-small cell lung, mutation, LKB1, EGFR, KRAS

## Abstract

Somatic mutations of *LKB1* tumour suppressor gene have been detected in human cancers including non-small cell lung cancer (NSCLC). The relationship between *LKB1* mutations and clinicopathological characteristics and other common oncogene mutations in NSCLC is inadequately described. In this study we evaluated tumour specimens from 310 patients with NSCLC including those with adenocarcinoma, adenosquamous carcinoma, and squamous cell carcinoma histologies. Tumours were obtained from patients of US (*n*=143) and Korean (*n*=167) origin and screened for *LKB1*, *KRAS*, *BRAF*, and *EGFR* mutations using RT—PCR-based SURVEYOR-WAVE method followed by Sanger sequencing. We detected mutations in the *LKB1* gene in 34 tumours (11%). *LKB1* mutation frequency was higher in NSCLC tumours of US origin (17%) compared with 5% in NSCLCs of Korean origin (*P*=0.001). They tended to occur more commonly in adenocarcinomas (13%) than in squamous cell carcinomas (5%) (*P*=0.066). *LKB1* mutations associated with smoking history (*P*=0.007) and *KRAS* mutations (*P*=0.042) were almost mutually exclusive with *EGFR* mutations (*P*=0.002). The outcome of stages I and II NSCLC patients treated with surgery alone did not significantly differ based on *LKB1* mutation status. Our study provides clinical and molecular characteristics of NSCLC, which harbour *LKB1* mutations.

Peutz–Jeghers syndrome (PJS) is caused by mutations in the *LKB1* tumour suppressor gene (Hemminki *et al*, 1998). LKB1 is serine–threonine kinase, which has been shown to regulate cell cycle progression, apoptosis, and cell polarity (Tiainen *et al*, 1999). The major target of LKB1 kinase activity is thought to be AMP-activated protein kinase (AMPK). AMPK is activated under low cellular energy conditions by raising AMP levels and it phosphorylates multiple downstream targets including tuberosclerosis complex 2 gene, which represses mTOR signalling. Phosphorylation of AMPK by LKB1 is needed for full activity of AMPK and suppression of mTOR activity under low energy conditions (Shaw *et al*, 2004). The hallmarks of PJS include mucocutaneous pigmentation and hamartomatous polyps of the gastrointestinal tract. Patients with PJS have an increased risk of developing gastrointestinal, pancreatic, breast, gynecological, and non-small cell lung cancers (NSCLC). The overall risk for cancers is increased 5- to 12-fold in different age groups compared with the general population (Hearle *et al*, 2006). Somatic mutations of the *LKB1* tumour suppressor have rarely been found in cancers from patients who do not have PJS except for NSCLC (Avizienyte *et al*, 1999). Previous reports have suggested the *LKB1* mutation rate to be as high as 30% in NSCLC tumours and cell lines derived from patients of Caucasian origin (Carretero *et al*, 2004; Matsumoto *et al*, 2007) and to be infrequent in NSCLC patients of Asian origin (3%) (Onozato *et al*, 2007). Furthermore, *LKB1* mutations have been shown to be associated with adenocarcinoma histology, male gender, and smoking history (Matsumoto *et al*, 2007). A recent report of using a mouse model for *lkb1* inactivation in NSCLC has provided insights into the role of the gene in this cancer. This study showed that *lkb1* inactivation in combination with activating mutations of *kras* using inducible promoters in the lung was associated with decreased survival compared with *kras* mutation alone (Ji *et al*, 2007).

Current screening techniques for *LKB1* tumour suppressor mutations rely on conventional exonic sequencing of the DNA, which can identify single base pair changes and small deletions/insertions (Ballhausen and Gunther, 2003). The addition of multiple ligation-dependent probe amplification (MLPA), which enables detection of exonic and whole gene deletions, with exonic sequencing has increased the mutation detection rates to 80% in patients with PJS phenotype (Volikos *et al*, 2006). Conventional sequencing has also been used to detect mutations of *LKB1* at mRNA level and some mutations missed by sequencing at the DNA level have been discovered by mRNA-based approaches (Abed *et al*, 2001). However, mutant forms of *LKB1* mRNAs can have a shortened half-life because of nonsense-mediated decay, which can potentially interfere with mutation detection (Abed *et al*, 2001).

We have recently described a rapid and sensitive enzymatic method to detect mutations in epidermal growth factor (*EGFR*) of DNA from fresh tissue and paraffin-embedded tissues (Janne *et al*, 2006). This method includes amplification of region of interest with PCR, SURVEYOR endonuclease digestion of the products, which cleaves mismatched heteroduplex DNAs, and detection of DNA fragments by sensitive high-performance liquid chromatography (HPLC) WAVE HS system. Subsequently, SURVEYOR-positive specimens are fractionated in partially denaturing conditions and are Sanger-sequenced. The major advantages of SURVEYOR-WAVE method are the fast exclusion of wild-type specimens without laborious conventional sequencing and high sensitivity. The SURVEYOR-WAVE method is more sensitive than conventional sequencing as it can detect mutant DNA sequences when they are present in 1% or more of total DNA (Janne *et al*, 2006).

The current study was designed to analyse the incidence of *LKB1* mutations in NSCLC. Furthermore, we wanted to investigate the *LKB1* mutational frequency in different histologies and ethnic backgrounds, and assess their correlation to smoking history, gender, stage, survival, and other oncogenic mutations in NSCLC.

## Materials and Methods

### Cell lines and tumour specimens

The NSCLC cell lines A549, NCI-H1395, NCI-H1650, NCI-H1666, NCI-H1781, NCI-1975, NCI-H23, NCI-H2126, NCI-H441, NCI-H820, HCC2935, HCC4006, and HCC827 were purchased from ATCC (Manassas, VA, USA). H3255, H3255GR, HCC2279, and PC-9 have been previously described (Ono *et al*, 2004; Tracy *et al*, 2004; Engelman *et al*, 2006). Ma1, and Ma70 are NSCLC cell lines harbouring *EGFR* mutations that were established at the Kinki University, Osaka, Japan. A549, NCI-H1395, NCI-H1666, NCI-H23, NCI-H2122, NCI-H2126, and NCI-H460 have previously been reported to contain *LKB1* mutations (Sanchez-Cespedes *et al*, 2002; Bamford *et al*, 2004; Carretero *et al*, 2004).

NSCLC tumours (*n*=310) were collected from surgical resections from patients with stages I–IV NSCLC when sufficient material for RNA extraction was available. The majority of the specimens (*n*=167) was collected at the Samsung Medical Center, Seoul, Korea. Frozen tumour tissues were collected from 809 out of 2442 patients who underwent curative resection for NSCLC from November 1995 to February 2007 at Samsung Medical Center. One or two pieces from the periphery of the tumour masses – avoiding necrotic regions – were immediately frozen at –80°C until retrieved. Medical records and haematoxylin and eosin-stained slides of the specimen were reviewed by a single pathologist. Only frozen tumour tissues from adenocarcinoma or squamous cell carcinoma (according to the 2004 World Health Organization histopathological criteria) were included. Only frozen tumour tissues with a tumour cell content of more than 70% were used for further analysis. In addition, frozen tumour tissues of the following patients were excluded from the study: patients who had received preoperative neoadjuvant treatments, patients with double primary lung cancer, and patients who had undergone incomplete resections or who had not been subjected to mediastinal lymph node dissections. Selected frozen tumour tissues were used for the microdissection. Briefly, frozen tissues were lightly stained with haematoxylin–eosin to improve visualisation, and necrotic tumour tissues and intervening normal tissues were removed. Each of the microdissected tumour tissues with a tumour cell content of more than 90% was placed in 1 ml Easy Blue reagent of a commercially available RNA isolation kit (easy-spin™ Total RNA Extraction Kit, iNtRON Biotechnology, Gyeonggi-do, Korea), immediately homogenised by vortexing, and the total RNA was extracted. The quantity and quality of RNA were analysed using a spectrometer (Nanodrop Technologies, Rockland, DE, USA) and Agilent 2100 Bioanalyzer (Agilent RNA 6000 Nano Kit, Agilent Technologies Inc., Böblingen, Germany), respectively. Finally, 167 frozen tissues with acceptable quality of RNA (RNA Integrity Number (RIN) value over 7.0) were used for the current studies. All patients provided written informed consent.

The tumours from Caucasian patients (*n*=143) were collected at the Brigham and Women's Hospital, Boston, MA, USA between 1991 and 1997 and have been previously published for patient characteristics and histology, and for expression profile-based clustering of the tumours (Bhattacharjee *et al*, 2001; Hayes *et al*, 2006). Frozen samples of resected lung tumours were obtained within 30 min of resection and subdivided into 100 mg samples and snap frozen at −80°C. Each specimen was associated with an immediately adjacent sample embedded for histology in an optimal cutting temperature medium and stored at −80°C. Six micrometres of frozen sections of embedded samples stained with haematoxylin and eosin were used to confirm the postoperative pathological diagnosis and to estimate the cellular composition of adjacent samples. All specimens underwent pathological review by two pathologists. In all 109 tumours obtained during the same time period were excluded because they did not meet one or more of the eligibility criteria. Tissue samples were homogenised in Trizol (Life Technologies, Gaithersburg, MD, USA) and RNA was extracted and purified by using the RNeasy column purification kit (Qiagen, Chatsworth, CA, USA). Denaturing formaldehyde gel electrophoresis followed by northern blotting using a *β*-actin probe assessed RNA integrity. Samples were excluded if *β*-actin was not full length. All patients provided written informed consent. The US cohort included specimens that have previously undergone analyses and the results have been published for *EGFR, KRAS*, and *BRAF* mutations (Bhattacharjee *et al*, 2001; Naoki *et al*, 2002; Hayes *et al*, 2006). We reconfirmed the mutations in 30 of these specimens using the SURVEYOR-based analysis (see section SURVEYOR digestion and HPLC analysis) and found 100% concordance between the two methods (data not shown).

Cell line specimens were snap frozen and stored at –80°C. RNA was extracted from tumours and cell lines using Trizol (Invitrogen, Carlsbad, CA, USA), purified with Rneasy Mini Kit (Qiagen, Valencia, CA, USA) and was used for cDNA synthesis using the QuantiTect reverse transcription kit (Qiagen, Valencia, CA, USA).

### PCR primers and cycling conditions

For *LKB1* gene analysis, PCR primers were designed to amplify the cDNA in two amplicons. PCR primers of the first amplicon were designed to hybridise to the noncoding area of the mRNA upstream of exon 1 (5′-agggaagtcggaacacaagg-3′) and to exon 5 (5′-ccagatgtccaccttgaagc-3′) generating a PCR product of 797 bp. The primers for the second amplicon located at exon 5 (5′-aacggcctggacaccttct-3′) and to noncoding exon 10 (5′-gaaccggcaggaagactgag-3′) generating a product of 702 bp, which has an overlapping part with first amplicon. For SURVEYOR-WAVE analysis of *KRAS*, PCR primers (5′-ggcctgctgaaaatgactga-3′, 5′-tcctgagcctgttttgtgtct-3′) were designed to generate an amplicon of 407 bp covering codons 12, 13 and 61, which are the codons commonly mutated in lung cancers. For SURVEYOR-WAVE mutation analysis of *BRAF*, cDNA was amplified in two overlapping amplicons (5′-aggatttcgtggtgatggag-3′, 5′-gatgacttctggtgccatcc-3′, and 5′-gacgggactcgagtgatgat-3′, 5′-ggtatcctcgtcccaccata-3′) covering codons 387–673. For SURVEYOR-WAVE analysis of *EGFR*, PCR amplification was done in a single amplicon (5′-ggagcctcttacacccagtg-3′, 5′-aggtcatcaactcccaaacg-3′), which covered exons 18–21 of the gene. PCR amplification was done using JumpStart Taq (Sigma, St Louis, MO, USA) under the manufacturer's guidelines. A part of the specimens (*n*=103) was previously characterised for *KRAS, BRAF, EGFR* mutations using reverse transcritase (RT)–PCR and direct sequencing of the PCR products (Naoki *et al*, 2002; Hayes *et al*, 2006).

### SURVEYOR digestion and HPLC analysis

SURVEYOR digestion and HPLC analysis were carried out as described previously (Janne *et al*, 2006). In brief, PCR products were digested in reaction mixture containing equal volumes of SURVEYOR enzyme (Transgenomics, Omaha, NE, USA) and Enhancer (Transgenomics, Omaha, NE, USA) at 42°C for 20 min followed by termination of the reaction by Stop Solution (Transgenomics, Omaha, NE, USA). Specimens were then loaded to the WAVE HS HPLC (Transgenomics, Omaha, NE, USA) at 50°C, eluted with an increasing acetonitrile gradient, and detected by UV detector using DNA intercalating fluorescence dye (Transgenomics, Omaha, NE, USA). When cell lines known to be homozygous for specific mutation were analysed, PCR products were mixed 1 : 1 with PCR products of a wild-type cell line, denatured by heating, and slowly renatured to generate heteroduplexes.

### Sequencing and fractionation

Specimens that showed an altered pattern on the SURVEYOR tracings were purified using QIAquick kit (Qiagen, Valencia, CA, USA) and sequenced bi-directionally by molecular biology core facility of Dana–Farber Cancer Institute. If a specimen showed an altered pattern on the SURVEYOR tracing but had a wild-type sequence by direct DNA sequencing, it underwent fractionation by WAVE HS HPLC in partially denaturing conditions. Running temperatures for specific amplicons were calculated by the Navigator Software (Transgenomics, Omaha, NE, USA). Collected fragments were amplified with PCR using the same primers as in the original amplification, purified and sequenced as previously described above.

### Statistical analysis

Fisher's exact test was used to assess the association of *LKB1* mutation status with other clinical, pathological, and genetic characteristics. To adjust for any difference between ethnic groups, the association between *LKB1* mutation rate and each characteristic was also evaluated as stratified contingency tables. If we did not reject that the odds ratios were the same across ethnic groups, we then tested whether the common odds ratios were unity based on the stratified Mantel–Haenszel estimate (Breslow and Day, 1980). Overall survival was estimated using the Kaplan–Meier method, with differences between the groups compared using the log-rank test. All *P*-values were based on a two-sided hypothesis, with *P*<0.05 considered to be statistically significant and 0.05<*P*<0.10 considered to be borderline significant.

## Results

### SURVEYOR-WAVE mutation detection of *LKB1* tumour suppressor in NSCLC cell lines

**Figure 1:**
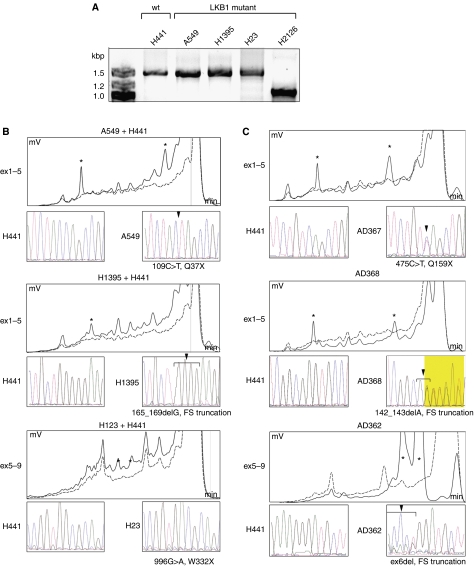
Mutation analysis of *LKB1* gene in NSCLC cell lines and tumours. RT–PCR amplification of cDNA from *LKB1* wt (H441) and *LKB1* mutant (A549, H1395, and H23) cell lines display the full length LKB1 mRNA (1.4 kbp) while the *LKB1* mutant cell line, H2126 with a deletion of exons 4–6 expresses a shorter mRNA (1.0 kbp) (**A**). HPLC tracings of SURVEYOR-WAVE mutation analysis of NSCLC cell lines A549, H1395, or H23 (continuous line), and H441 (dashed line). Time in minutes is shown on the *X*-axis, voltage in mV on the *Y*-axis (**B**). A549 and H1395 show novel peaks (^*^) in the amplicon covering exons 1–5 (ex1–5) corresponding to 109C>T, Q37X and 165_169delG, frameshift and truncation (FS truncation) mutations. The analysis from H23 demonstrates novel peaks in the amplicon covering exons 5–9 (ex5–9) corresponding to 996G>T, W332X mutation. *LKB1* wild-type cDNA (H441) was added to PCR products 1 : 1, denatured by heating and slowly renaturated to generate heteroduplexes since A549, H1395, and H23 have previously reported to be homozygous for the *LKB1* mutations. SURVEYOR-WAVE mutation analyses of NSCLC tumours (**C**). AD367 and AD368 tumours showed novel peaks in the ex1–5 amplicon corresponding to 475C>T, Q159X, and 142_143delA, FS, truncation mutations. AD362 tumour had novel peaks in ex5–9 amplicon corresponding to deletion of exon 6. Mutant sequences for AD367 and AD368 are displayed from sequences using the forward primer while mutation of the AD362 is showed with reverse primer.

The impact of the stability of *LKB1* mRNA on detecting *LKB1* mutations was tested using RT–PCR with mRNA extracted from NSCLC cell lines that had previously been characterised for *LKB1* mutations. These included NCI-H441 (wild type) and A549, NCI-H1395, NCI-H23, and NCI-H2126 (all containing *LKB1* mutations). Reverse transcriptase–PCR amplification of the whole coding region of the *LKB1* mRNA showed that cell lines with nonsense (A549, NCI-H23) mutations or 1 bp deletion (H1395) expressed mRNA with comparable size to the wild-type H441 cell line (1460 bp). H2126 cell line, which is known to have homozygous deletion of exons 4–6, expressed mRNA with substantially smaller size (∼1000 bp) corresponding to deletion of 398 bp. RT–PCR revealed no major difference in *LKB1* mRNA expression levels between *LKB1* mutant or wild-type cell lines (Figure 1A).

As *LKB1* mutant and wild-type cell lines expressed comparable amounts of *LKB1* mRNA with RT–PCR, we studied the cDNA for mutations using the SURVEYOR-WAVE method. The WAVE HPLC provides a system to analyse DNA fragments smaller than 900 bp and therefore we designed two overlapping amplicons covering exons 1–5 (797 bp) and 5–9 (702 bp) to amplify the whole coding region of *LKB1* mRNA. PCR products of *LKB1* mutant cell lines were mixed 1 : 1 with the products from wild-type cell lines (H441) to generate heteroduplexes as *LKB1* mutant cell lines were previously reported to be homozygous for the inactivation of the gene. SURVEYOR-WAVE analysis of the amplicon covering exons 1–5 revealed novel peaks with the cDNA for A549, and NCI-H1395 cell lines compared with the wild type from NCI-H441 (Figure 1B). SURVEYOR-WAVE analysis of exons 5–9 showed novel peaks for the NCI-H23 cell line as well. The mutations detected with SURVEOR-WAVE were confirmed by conventional DNA sequencing and they corresponded to previous reports (Sanchez-Cespedes *et al*, 2002; Carretero *et al*, 2004). We could not detect the *LKB1* mutation of H2126 cell line with SURVEYOR-WAVE method using a two-amplicon approach because this cell line has a homozygous deletion of exons 4–6 and the reverse primer of the first amplicon and the forward primer of the second amplicon, which lie on the deleted part of the gene (data not shown).

### *LKB1* tumour suppressor gene mutations in NSCLC tumours

We next used the SURVEYOR-WAVE method to screen NSCLC tumour specimens (*n*=310) for *LKB1* mutations. We detected 34 *LKB1* mutations (11%) in the NSCLC tumour specimens (Table 1). The majority of the *LKB1* mutations detected was deletions or insertions (*n*=25, 74%). The remainder was missense (*n*=7, 21%) and nonsense (*n*=2, 6%) mutations (Table 2, Figure 1C). About one-half of the deletions and insertions were small, covering <15 bp (*n*=14, 56%), whereas larger deletions (*n*=11, 44%) covering hundreds of base pairs were detected in the remaining specimens. Some mutational hotspots were discovered. The areas that had the same mutation in more than one tumour specimen included deletion of exon 4 (*n*=4), deletion of exons 2 and 3 (*n*=3), D194Y (*n*=2), and P281L (*n*=2). Interestingly, a significant portion of the mutations was located in exon 1 (*n*=11, 32%) but there was no area of recurrent mutations in this exon (Table 2). Of the missense mutations detected in the current study, all except R426W are in the kinase domain of the protein. Missense mutations in codons 176 and 194 have been previously characterised in PJS (Launonen, 2005). We also found four F354L alterations (data not shown) but we did not consider these as missense mutations as this alteration has previously been reported to be a rare polymorphism of the gene (Launonen *et al*, 2000). We did not have access to the corresponding normal tissues and therefore, we could not verify if some of the missense mutations were somatic or germline.

### Association of *LKB1* tumour suppressor mutations in NSCLC with clinicopathological characteristics

**Figure 2:**
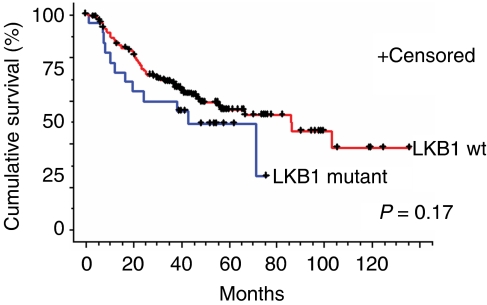
Kaplan–Meyer survival curves of stage I and II NSCLC patients with LKB1 wildtype (red line, *n*=198) *vs* LKB1 mutant (blue line, *n*=23) tumours.

The mutation frequency of *LKB1* gene was significantly higher in NSCLCs in the Caucasian cohort (Table 1). Twenty-five (17% of specimens) of the *LKB1* mutations were detected in NSCLCs collected from patients in the United States, whereas only nine mutations (5% of specimens) were detected in the Korean cohort (*P*=0.001) (Table 1). The *LKB1* mutation rate tended to be higher in adenocarcinomas (13%) compared with squamous cell carcinomas (5%) (*P*=0.067). Differences in histological subgroups were relatively modest in the US cohort with mutations in 18 out of 94 (19%) adenocarcinomas *vs* 5 out of 38 (13%) in squamous cell cancers (*P*=0.461). This is in contrast to the findings in the Asian patients where all of the *LKB1* mutations were detected in adenocarcinomas (9 out of 113 (8%)) and none were detected in squamous cell cancers (0 out of 54 (0%); *P*=0.032). Nevertheless, the higher rate of *LKB1* mutation in adenocarcinomas compared with squamous cell carcinomas retains the same level of statistical significance (stratified *P*=0.064) after adjusting for fluctuation between ethnic groups. The US cohort also included nine specimens from adenosquamous carcinomas and two out of nine (22%) had *LKB1* mutations, which is similar to the frequency in adenocarcinomas in this population (Table 1). There was no association between *LKB1* mutations and the clinical stage of the NSCLC patients. Kaplan–Meier survival curves of stages I and II NSCLC patients showed a tendency for shorter survival in patients with *LKB1* mutant tumours but this, however, did not reach statistical significance (*P*=0.17) (Figure 2). No differences in survival were observed in patients who harboured both *LKB1* and *KRAS* mutations compared with those with *KRAS* or *LKB1* alone but the total number of patients with both mutations who had stages I or II NSCLC was small (*n*=9; data not shown). We detected an association of *LKB1* mutations with a smoking history (*P*=0.007) and only two mutations were detected in tumours from 72 NSCLC patients who were either never or light (⩽10 pack years) former smokers (Table 1). After adjusting for ethnic group, the higher rate of *LKB1* mutation among patients with a smoking history is borderline significant (stratified *P*=0.067). The reduction in statistical significance is likely owing to the loss of power associated with the overall rarity of *LKB1* mutations among never or light former smokers. For these analyses we combined both never smokers and light (⩽10 pack years) smokers as the frequency of mutations in other oncogenes such as *EGFR* is similar in these two patient groups (Pham *et al*, 2006). There were no correlations between *LKB1* mutations and gender or age of a patient.

### Association of *LKB1* mutations with *K-Ras*, *B-Raf*, and *EGFR* mutations in NSCLC

Previous reports have suggested that in NSCLC cell lines, *LKB1* mutations often occur concurrently with *KRAS* or *BRAF* mutations (Sanchez-Cespedes *et al*, 2002; Carretero *et al*, 2004). Furthermore, *EGFR* mutations are often mutually exclusive with *KRAS* mutations in NSCLC (Kosaka *et al*, 2004; Marchetti *et al*, 2005). We used combined data from previous papers (Sanchez-Cespedes *et al*, 2002; Carretero *et al*, 2004) and from Sanger institute's databases (Bamford *et al*, 2004) to analyse association of *LKB1* mutations with mutations of *KRAS*, *BRAF*, and *EGFR*. Analysis of *LKB1* mutation harbouring NSCLC cell lines (A-427, A549, NCI-H1395, NCI-H1666, NCI-H2122, NCI-H2126, NCI-H23, and NCI-H460) showed that five of the cell lines (63%) had concurrent *LKB1* and *KRAS* mutations, two (25%) had concurrent *LKB1* and *BRAF* mutations, and only one (13%) had neither *KRAS* nor *BRAF* mutations. None of these cell lines had EGFR mutations.

As our findings in NSCLC cell lines suggested concurrency of *KRAS* or *BRAF* and mutual exclusiveness of *EGFR* mutations with LKB1 mutations, we analysed the mutational status of these genes in our primary NSCLC tumour specimens. *KRAS* mutations were detected in 49 (16% in the whole tumour set, 25% in Caucasian and 8% in Asian specimens) tumour specimens with 10 (20% of *KRAS* mutants) of these occurring concurrently with an *LKB1* mutation (*P*=0.042) (Table 3). Four *BRAF* mutations (1%) were found in the tumour set (G465V, N581S, L596R, and T599I) and one of these (N581S) occurred concurrently with *LKB1* mutation (*P*=0.373). Seventy tumours (23% in the whole tumour set, 9% in Caucasian, 34% in Asian specimens) had *EGFR* kinase domain mutations with only one of them occurring concurrently with an *LKB1* mutation (*P*=0.002). The tumour with a concurrent *EGFR* and *LKB1* mutation had a missense mutation of *LKB1* outside the kinase domain (R426W). No germ line DNA was available from this patient. However, a recent report has suggested that R426W is in fact a rare polymorphism of the gene (Onozato *et al*, 2007). Taken together our findings suggest that unlike *KRAS*, mutations in *EGFR* and *LKB1* are mutually exclusive in NSCLC.

Previous reports (Sanchez-Cespedes *et al*, 2002; Carretero *et al*, 2004) and Cancer Genome Project by Sanger Institute (Bamford *et al*, 2004) have extensively characterised *LKB1* mutations in NSCLC cell lines with *KRAS* and *BRAF* mutations, but *LKB1* status of *EGFR* mutant NSCLC cell lines has not been extensively analysed. Therefore, we analysed the *LKB1* genotype of NSCLC cell lines with known *EGFR* or *ERBB2* mutations. Twelve *EGFR* mutant and one *ERBB2* mutant cell lines were analysed for *LKB1* genotype. No *LKB1* mutations were detected in these cell lines (Table 4).

## Discussion

The present study characterised *LKB1* mutation frequency in NSCLC using one of the largest tumour sets to date (*n*=310). Our study analysed tumours from different histologies and of both a US and Korean origin to determine potential histological and ethnic variation in *LKB1* mutational frequency. The large size of our study enabled us to study associations of *LKB1* mutations with clinocopathological factors, which have been incompletely characterised in previous studies (Sanchez-Cespedes *et al*, 2002; Carretero *et al*, 2004; Fernandez *et al*, 2004; Matsumoto *et al*, 2007; Onozato *et al*, 2007). In addition, we used a modification of a sensitive mutation screening technique that we have previously developed to facilitate the rapid detection of *LKB1* mutations (Janne *et al*, 2006).

Findings from our study confirm the high frequency of *LKB1* mutations in NSCLC (11%), which in contrast, are rare (0–4%) in other common solid malignancies (Avizienyte *et al*, 1998, 1999). The reason behind these observations is presently unknown but might reflect the differences in carcinogen exposure in the lungs compared with other tissues. In support of this hypothesis, we find that *LKB1* mutations are significantly (*P*=0.007) more common in smokers than in never or light (⩽10 pack years) cigarette smokers (Table 1). Male PJS patients (age ⩾50 years) have an increased risk of developing lung cancer compared with the general population but the relationship of smoking and the increased risk of lung cancer in PJS is unknown (Hearle *et al*, 2006). Interestingly, the *LKB1* mutation spectrum found in the current study is very similar to those previously published for PJS (deletions 34%, insertions 15%, splice site mutations 14%, missense mutations 21%, and nonsense mutations 12%) (Launonen, 2005) and, as in PJS, no clear mutational hotspots were detected.

Our study also demonstrated that the *LKB1* mutation frequency was significantly higher in cancers derived from a US population compared with those found in Korean patients (17 *vs* 5%; *P*=0.001). These differences also track with cigarette smoking, as the number of never/light former smokers was much higher in the Korean cohort compared with the US cohort of patients (38 *vs* 13%). Similarly, a recent study of 100 Japanese NSCLCs found that only 3% contained an *LKB1* mutation (Onozato *et al*, 2007). These findings are in contrast to *EGFR* mutations, which are more frequently detected in tumours from never/light cigarette smokers and from Asian patients (Janne and Johnson, 2006). Our studies further highlight ethnic and environmental differences in the origins of NSCLC.

Given the differences in *LKB1* mutation frequencies in smokers *vs* never/light smokers and in the US compared with Korean patients, we further determined whether these were also associated with other oncogene mutations known to vary in these subgroups of patients. Consistent with prior studies we found a significant association with concurrent *KRAS* mutations, which are common in smokers (Ahrendt *et al*, 2001), in one out of three of NSCLC with *LKB1* mutations (Table 3). In contrast, there was a significant inverse relationship of *LKB1* mutations with *EGFR* mutations in both NSCLC tumours and cell lines, which has not previously been described (Tables 3 and 4). These differences may relate to the biological role of LKB1 in lung cancer. It is possible that in *EGFR* mutant lung cancers there is already maximal activation of the PI3K/Akt/mTOR signalling pathway and thus an *LKB1* mutation may not be required to further potentiate this signalling pathway. In contrast in *KRAS* mutant cancers, a concurrent *LKB1* mutation may be required to enhance mTOR activation. Mice with concurrent *KRAS* mutations and *LKB1* inactivation have more aggressive tumours and a shorter survival than those with only *KRAS* mutant cancers (Ji *et al*, 2007). In our study, we were not able to detect a significant survival difference for patients whose tumours contained *LKB1* mutations alone or concurrently with *KRAS* mutations (data not shown) likely because of the limited number of tumour specimens. Additional studies are needed to clarify the prognostic impact of *LKB1* mutations in humans with NSCLC. In the present study ∼2 out of 3 of *LKB1* mutant tumours were *KRAS* wild type (Table 3). One possibility is that such tumours contain a concurrent mutation in another oncogene that activates the same signalling pathway as *KRAS.* For this reason, we examined our tumours for *BRAF* mutations, which are found in 1–2% of NSCLC (Naoki *et al*, 2002). We detected a concurrent *LKB1* mutation in one of the four *BRAF* mutant tumours (Table 3). This tumour was wild type for *KRAS* (data not shown). In addition, some of the *BRAF* mutant NSCLC cell lines (NCI-H1395, G469A; NCI-H1666, G466V) also contain a concurrent *LKB1* mutation (Sanchez-Cespedes *et al*, 2002; Bamford *et al*, 2004; Carretero *et al*, 2004). Future studies will help further clarify whether *LKB1* mutations occur concurrently with other genomic alterations in NSCLC and the impact of this on patient outcome.

Our study employed a mutation scanning technology to screen for *LKB1* mutations at the cDNA level (Janne *et al*, 2006). This was advantageous as the entire coding region of *LKB1* could be rapidly screened for a mutation using just two overlapping cDNA fragments. *LKB1* is a challenging gene to analyse at the genomic DNA level because of its high guanine–cytosine content. In addition, as many of the *LKB1* mutations are small deletions (Table 2) or involve deletions of entire exons, these would be missed using exon-specific genome sequencing methods. Our method, however, does have limitations as it would miss deletions at the site of PCR primers, whole gene deletions, or deletions within the promoter region all of which have been infrequently detected in PJS (Volikos *et al*, 2006). Thus our studies may underestimate the true *LKB1* mutation frequency in NSCLC. In addition, our method is limited to the analysis of fresh tumour specimens, which are available only from the minority of NSCLC patients. Furthermore, as techniques isolating mRNA from formalin-fixed paraffin-embedded tumour specimens continue to improve, this rapid mutation scanning technique can be used to analyse broader populations of tumours from NSCLC patients. Future studies may need to employ a combination of *LKB1* mutation detection methodologies including the current method, MLPA and direct sequencing.

## Figures and Tables

**Table 1:** Frequency of *LKB1* mutations in NSCLC tumours and their association with clinicopathological characteristics

	**LKB1 mutation**		
	+	−	*P*-value*
All tumours	34 (11%)	276 (89%)	
Age, median	61.2	62.2	
			
*Ethnicity*			
Caucasian cohort	25 (17%)	118 (83%)	0.001
Asian cohort	9 (5%)	158 (95%)	
			
Gender			
Male	20 (11%)	167 (89%)	NS
Female	14 (12%)	107 (88%)	
			
*Smoking*			
Never (<10 py)	2 (3%)	70 (97%)	0.007
Smoker (>10 py)	26 (14%)	161 (86%)	
			
*Tumour stage*			
I	19 (10%)	169 (90%)	NS
II	8 (14%)	51 (86%)	
III	5 (11%)	42 (89%)	
IV	1 (12%)	7 (88%)	
			
*Histology*			
Adenocarcinoma	27 (13%)	180 (87%)	0.047
Squamous carcinoma	5 (5%)	87 (95%)	
Adenosquamous	2 (22%)	7 (78%)	

^*^Fisher's exact test, NS=not statistically significant (*P*>0.05).

**Table 2:** The specific *LKB1* mutations in NSCLC tumours

**Mutation type**	**No. (%)**	**Mutation**	**Amino acid change**	**Exon**	**Histology**
Missense	7 (21)	a526G>T	D176Y	4	Ad
		a580G>T	D194Y	4	Ad
		580G>T	D194Y	4	Sq
		829G>T	D277Y	6	AdSq
		a842C>T	P281L	6	Ad
		a842C>T	P281L	6	Ad
		1276C>T	R426W	9	Ad
Nonsense	2 (6)	206C>A	S69X	1	Ad
		475C>T	Q159X	4	Ad
Deletion/ insertion	25 (74)	a75_76del2&insT	FS, truncates	1	Ad
		120_130del11	FS, truncates	1	Ad
		125_127insGG	FS, truncates	1	Ad
		128_129delC	FS, truncates	1	Ad
		142_143delA	FS, truncates	1	Ad
		180delC	FS, truncates	1	Ad
		209delA	FS, truncates	1	Ad
		227_228delC	FS, truncates	1	Ad
		47_651del604	FS, truncates	1-5	Sq
		153_536del384	FS, truncates	1-4	AdSq
		aexon 2-3del	Truncates	2-3	Sq
		aexon 2-3del	Truncates	2-3	Ad
		exon 2-3del	Truncates	2-3	Ad
		exon 2-4del	FS, truncates	2-4	Sq
		464_465del2insTTTGCT	FS, truncates	3-4	Sq
		562_563delG	FS, truncates	4	Ad
		aexon 4del	FS, truncates	4	Ad
		exon 4del	FS, truncates	4	Ad
		exon 4del	FS, truncates	4	Ad
		exon 4del	FS, truncates	4	Ad
		610_623del14	FS, truncates	5	Ad
		a837_844delC	FS, truncates	6	Ad
		837_844insC	FS, truncates	6	Ad
		exon 6del	FS, truncates	6	Ad
		1038_1040insG	FS, truncates	8	Ad

Ad=Adenocarcinoma; AdSq=Adenosquamous carcinoma; Sq=Squamous cell carcinoma; .

a These mutations were detected in Korean NSCLC patients.

**Table 3:** Association of *LKB1* mutations with *KRAS*, *BRAF,* and *EGFR* mutations in NSCLC tumours

		***LKB1* mutation**		
		+	−	*P*-value*
*EGFR* mutation	+	1	69	0.002
	−	33	207	
*K-Ras* mutation	+	10	39	0.042
	−	24	237	
B-Raf *mutation*	+	1	3	0.373
	−	33	273	

^*^Fisher's exact test.

**Table 4:** LKB1 genotypes of NSCLC cell lines with EGFR or ERBB2 mutations

**Cell line**	***EGFR* genotype**	***HER2* genotype**	***LKB1* genotype**
H1650	E746_A750del	Wt	Wt
H1781	Wt	G776V, Cins	Wt
H1975	L858R, T790M	Wt	Wt
H3255	L858R	Wt	Wt
H3255GR	L858R, T790M	Wt	Wt
H820	L747_L751del, T790M	Wt	Wt
HCC2279	E746_A750del	Wt	Wt
HCC2935	E746_T751del, S752I	Wt	Wt
HCC4006	L747_E749del, A750P	Wt	Wt
HCC827	E746_A750del	Wt	Wt
Ma-1	E746_A750del	Wt	Wt
Ma-70	L858R	Wt	Wt
PC-9	E746_A750del	Wt	Wt

Wt=wild type.
